# Enhanced production of berberine in In vitro regenerated cell of *Tinospora cordifolia* and its analysis through LCMS QToF

**DOI:** 10.1007/s13205-016-0592-6

**Published:** 2017-04-11

**Authors:** Jitendra Mittal, Madan Mohan Sharma

**Affiliations:** Department of Biosciences, Manipal University Jaipur, VPO Dehmikalan, Jaipur Ajmer Expressway, Jaipur, Rajasthan India

**Keywords:** Cytokinins, Auxins, ISSR, Berberine, UPLC, QToF

## Abstract

*Tinospora cordifolia* is a prioritized medicinal plant and having an immense medicinal importance especially in Indian medicinal system. But this plant needs a regeneration protocol for its rapid propagation. An efficient regeneration protocol was developed for *T. cordifolia* using nodal explants. High frequency of multiple shoot formation was induced when the nodal segments were cultured on MS medium supplemented with BAP (1.0 mg L^−1^) and 2-iP (0.5 mg L^−1^). The highest mean number of shoots per nodal explant (7.9 ± 0.45) with highest shoot length (9.3 ± 0.48 cm) and 86% response were achieved on this media and hormonal concentration. The optimum rooting was obtained on ½ strength of MS medium augmented with IBA (0.5 mg L^−1^) with 8.3 ± 0.46 cm root length and 89% response. Micropropagated plantlets were found to be identical with the mother plant when clonal fidelity of these plantlets were analyzed with inter simple sequence repeat (ISSR) marker. The berberine content was analyzed through LCMS QToF and the highest amount was found in in vitro callus (19.8 µg/gm) followed by stem (9.3 µg/gm) and leaves of field-grown plants (8.4 µg/gm). Further, presence of berberine was confirmed by ESI–MS spectra with protonated molecular ions ([M + H]^+^) at *m/z* 336. Furthermore, MS–MS fragmentation pattern confirmed for the presence of berberine in both the samples. Both the spectra (standard and samples) showed common peaks for berberine in the form of protonated molecular ions ([M + H]^+^) at *m/z* 320, *m/z* 304, *m/z* 292, *m/z* 278 in MS/MS mode. The study revealed that developed protocol is potent for rapid mass propagation of this plant species with high accumulation of important secondary metabolite berberine.

## Introduction

*Tinospora cordifolia* (Willd.) Miers ex Hook. F. & Thoms is an important medicinal climber, found in tropical regions of India, China, Sri Lanka, and Bangladesh (Mittal et al. 2014). Besides, it is rich with a variety of natural chemical constituent’s viz., tinosporin, cordifolioside, magnoflorine, palmetine, isocolumbin, tinocordiside, glycoside, cordifolioside syringing (Nagarkar et al. 2013; Choudhry et al. 2014) and cures a number of ailments such as viral infections, cancer, diabetes, inflammation, neurological disorders, psychiatric problems, microbial infection, hyper tension and HIV aids (Jayaganthan et al. 2013; Nagarkar et al. 2013; Joladarash et al. 2014; Mittal et al. 2014). Recently, this plant species is extensively using to cure chikungunya and dengue. Many ayurvedic pharmaceutical industries are producing medicines using Giloy to cure these diseases.

Further, medicinal plants are becoming endangered at an increasing rate owing to urbanization, deforestation and uprooting of complete plants to procure plant extract for the production of medicines by pharmaceutical companies (Mohammed and Kumar 2012). Consequently, medicinal flora is decreasing at a faster rate from their natural habitat. Similarly, *Tinospora cordifolia* is an overexploited plant and listed in prioritized medicinal plant list by national medicinal plant board (NMPB) Govt. of India (Raghu et al. 2006; Kala and Sajwan 2007). Overexploitation of this plant species has led to its acute scarcity to meet the present-day demand. Plant tissue culture fulfills one of this demand and conserve this NMPB prioritized plant. Previously, few reports are available regarding the micropropagation of *T. cordifolia* through nodal segments but the produced regeneration protocols revealed comparatively less number of shoots and are unreliable due to less number of in vitro shoot propagation (Kumar et al. 2003; Raghu et al. 2006; Gururaj et al. 2007; Khanapurkar et al. 2012; Sivakumar et al. 2014).

In this research work, we have reported a competent, reliable and reproducible protocol for the in vitro regeneration of *T. cordifolia* using mature nodal explants. To date, none of the reported micropropagation protocols has assessed the genetic fidelity of tissue culture raised plants through Inter Simple Sequence Repeat (ISSR) marker. This study revealed that the tissue culture raised plants were identical with the donor mother plant. Besides, the berberine analysis has also been done to compare the produced amount of berberine in the in vivo leaf, stem, aerial roots and in in vitro regenerated calli of *T. cordifolia* by using LC–MS QToF technology.

## Result and discussion

### Micropropagation

**Fig. 1:**
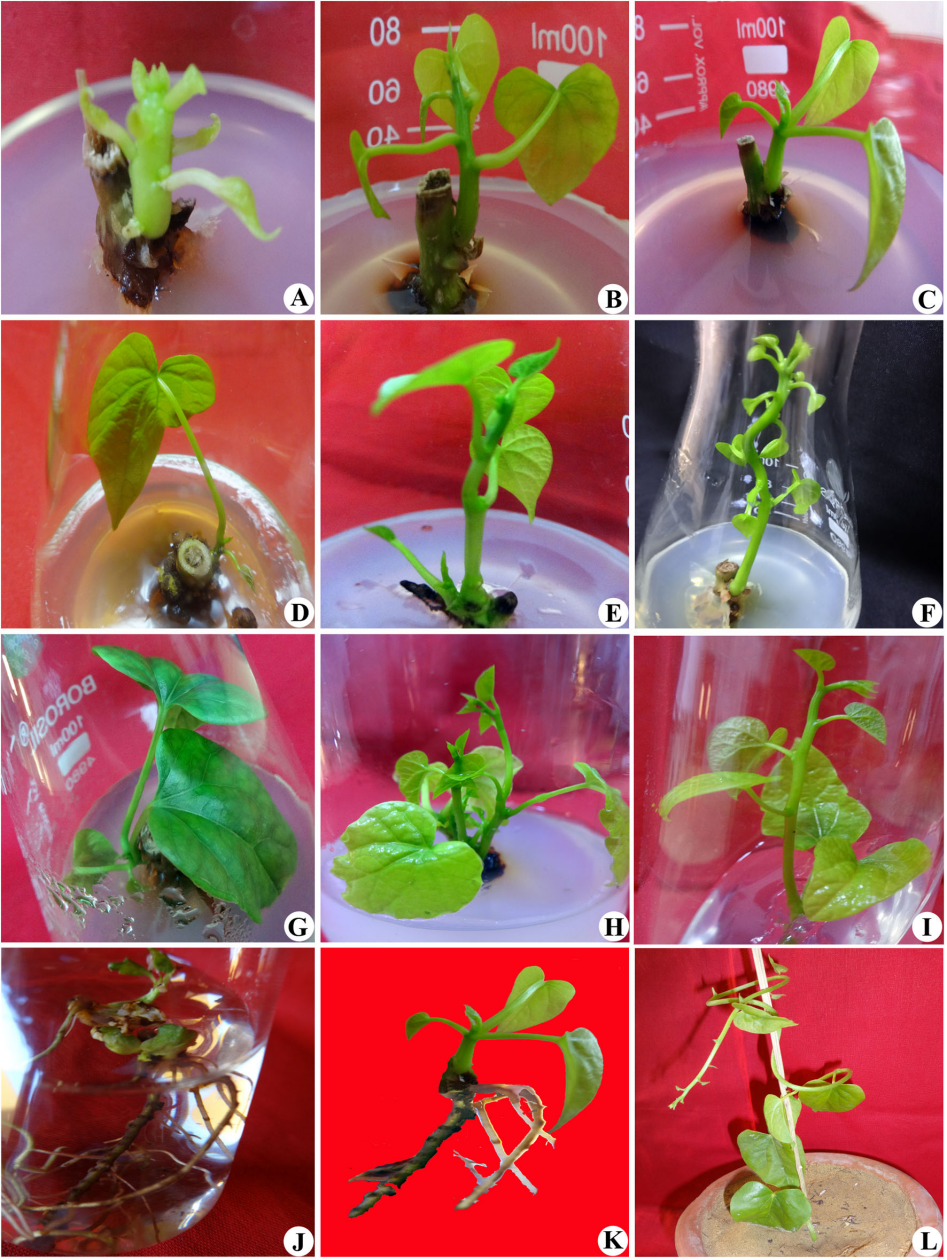
**a**–**l** Direct in vitro propagation of *T. cordifolia* through nodal segments; **a**, **b**: Effect of BAP (1 mg L^−1^) on axillary bud proliferation after 3 weeks of inoculation; **c**: Influence of Kn (1 mg L^−1^) on shoot bud proliferation 3 weeks of inoculation; **d**: Effect of TDZ (1 mg L^−1^) on shoot formation 3 weeks of inoculation; **e**: Influence of 2-iP (0.5 mg L^−1^) on shoot bud proliferation 3 weeks of inoculation; **f**: Synergistic effect of BAP (1 mg L^−1^) and Kn (1 mg L^−1^) on further differentiation of shoots 3 weeks of subculture; **g**: Combined effect of BAP (1 mg L^−1^) and TDZ (1 mg L^−1^) on further multiplication of shoots 3 weeks of subculture; **h**: Optimum multiplication of shoots on BAP (1 mg L^−1^) and 2-iP (0.5 mg L^−1^) after 3 week of subculture; **i**: Elongation of regenerated shoots after another 2 weeks; **j**: Induction of in vitro roots on half MS with IBA (0.5 mg L^−1^) after 2 weeks; **k**: Exposed view of complete plantlets; **l**: Hardened plant acclimatized in natural environment

In vitro regeneration was successfully achieved using mature in vivo nodal explant (1.0–1.5 cm long) of *T. cordifolia* through direct method of tissue culture (Fig. 1a–l). During the investigation, maximum numbers of shoots (3.9 ± 0.25) with 5.2 ± 0.41 cm shoot length were obtained on MS medium supplemented with BAP (1.0 mg L^−1^) (Table 1; Fig. 1a, b). Number of shoots as well as shoot length decreased as the concentration of BAP decreased/increased beyond 1.0 mg L^−1^ (Table 1). The multiplication of shoots using BAP has also been reported in *T. cordifolia* (Raghu et al. 2006) as well as in other medicinal plants such as *Portulaca oleracea*, *Asparagus racemosus* and *Cedrela fissilis* (Sharma et al. 2011; Thakur et al. 2015; Aragão et al. 2016) which supports the present results. In contrast to the above results, Faisal and Anis (2003) reported that Kn was optimum for in vitro shooting in *Tylophora indica*.

**Table 1:** Effect of cytokinin for multiplication of shoots through nodal explants in *T. cordifolia*

Cytokinin (mg L^−1^)				Shoot number with response	
BAP	Kn	2iP	TDZ	Shoot number ± S.D.	Response (%)
0.5	–	–	–	2.2 ± 0.41^e^	63
1.0	–	–	–	3.9 ± 0.25^c^	93
2.0	–	–	–	2.4 ± 0.48^e^	81
–	0.5	–	–	1.2 ± 0.25^efg^	59
–	1.0	–	–	1.4 ± 0.41^efg^	63
–	2.0	–	–	1.26 ± 0.36^efg^	48
–	–	0.5	–	2.8 ± 0.41^d^	72
–	–	1.0	–	2.0 ± 0.26^ef^	75
–	–	2.0	–	1.7 ± 0.45^efg^	70
–	–	–	0.5	1.9 ± 0.35^efg^	69
–	–	–	1.0	2.7 ± 0.45^d^	68
–	–	–	2.0	2.0 ± 0.25^ef^	69
1.0	0.5	–	–	2.1 ± 0.35^ef^	58
1.0	1.0	–	–	2.7 ± 0.61^d^	55
1.0	–	0.5	–	7.9 ± 0.45^a^	86
1.0	–	1.0	–	4.2 ± 0.41^b^	80
1.0	–	–	0.5	2.8 ± 0.41^d^	74
1.0	–	–	1.0	2.3 ± 0.45^e^	71

Different characters (a, b, c etc) are the analysis of variance observed in the group of data. It is a statistical analysis of data by t Test and ANOVA

However, in addition to BAP, Kn, TDZ and 2-iP at their varied concentrations revealed variable shooting response (Table 1; Fig. 1b–g). The reports from Lindiro et al. (2013) in *Chrysanthemum cinerariaefolium* and Kher et al. (2014) in *Pluchea lanceolata* supported this result. In divergence to the reports on TDZ and 2-iP, Kn in *Matthiola incana* (Hesar et al. 2011) and BAP with NAA in *Vigna subterranea* (Kone et al. 2013) have also proved better for the multiplication of shoots.

Optimized concentration of BAP (1.0 mg L^−1^) when combined with optimized concentration of 2-iP (0.5 mg L^−1^) gave maximum number of shoots (7.9 ± 0.45) (Table 1; Fig. 1h, i). Moreover, the length of shoots was higher on the same medium with hormones utilized for the multiplication of shoots in previous experiment (Fig. 1h). Similar response of BAP in combination with 2-iP for shoot multiplication has also been reported in *Croton scabiosus* (Salamma and Rao 2014). On contrary to the above result, optimum shoot multiplication was obtained on MS medium fortified with a combination of BAP and Kn in *Momordica balsamina and T. cordifolia* (Thakur et al. 2011; Sivakumar et al. 2014), TDZ and NAA in *Melastoma malabatricum* (Ghimire et al. 2016). In vitro raised elongated shoots (9.3 ± 0.48 cm) were transferred onto root induction medium composed of full, ½ and ¼ strength of MS salts. Only half strength of MS medium fortified with IBA (0.5 mg L^−1^) gave optimum rooting (Table 2; Fig. 1j, k). IAA (0.1–1.0 mg L^−1^) did not produce positive response for rooting. In vitro rooting on ½ MS medium supplemented with IBA is also supported by the earlier workers on the same hormone and medium in various plants *T. cordifolia*, *Dioscorea remotiflora* and *Leptadenia reticulate* (Gururaj et al. 2007; Bernabe-Antonio et al. 2012; Rathore and Shekhawat 2013). Higher concentration of IBA beyond 0.5 mg L^−1^ induced callusing at the lower end of stem and inhibited root growth in the culture. In contrast, IAA has also been reported for in vitro root formation in *T. cordifolia*, *Lathyrus sativus* and *Bacopa monneri* (Raghu et al. 2006; Barpete et al. 2014; Kapil and Sharma 2014).

**Table 2:** Effect of strength of MS salts with auxin for in vitro rooting in *Tinospora cordifolia*

Medium	IAA (mg L^−1^)	IBA (mg L^−1^)	Root length (cm)	Response (%)
Full MS	0.25	–	Callus	–
Full MS	0.5	–	Callus	–
Full MS	1.0	–	Callus	–
Full MS	–	0.25	Callus	–
Full MS	–	0.5	Callus	–
Full MS	–	1.0	Callus	–
½ MS	0.25	–	3.5 ± 0.53^c^	40
½ MS	0.5	–	3.8 ± 0.46^c^	46
½ MS	1.0	–	Callus	–
½ MS	–	0.25	5.4 ± 0.52^b^	68
½ MS	–	0.5	8.3 ± 0.46^a^	89
½ MS	–	1.0	Callus	–
¼ MS	0.25	–	3.0 ± 0.53^d^	49
¼ MS	0.5	–	2.9 ± 0.35^d^	41
¼ MS	1.0	–	Callus	–
¼ MS	–	0.25	3.6 ± 0.51^c^	61
¼ MS	–	0.5	3.9 ± 0.35^c^	58
¼ MS	–	1.0	Callus	–

Different characters (a, b, c etc) are the analysis of variance observed in the group of data. It is a statistical analysis of data by t Test and ANOVA

After 3 weeks of sub-culturing onto rooting medium, lateral roots were produced. The complete plantlets were transplanted ex vitro and raised in earthen pots (Fig. 1l) containing sterilized garden soil, and cocopeat (2:1). The hardening and acclimatization was done by the procedure mentioned in “Materials and methods”. Approximately, 70% of plantlets survived well when transferred in natural environment.

### Callus culture

**Fig. 2:**
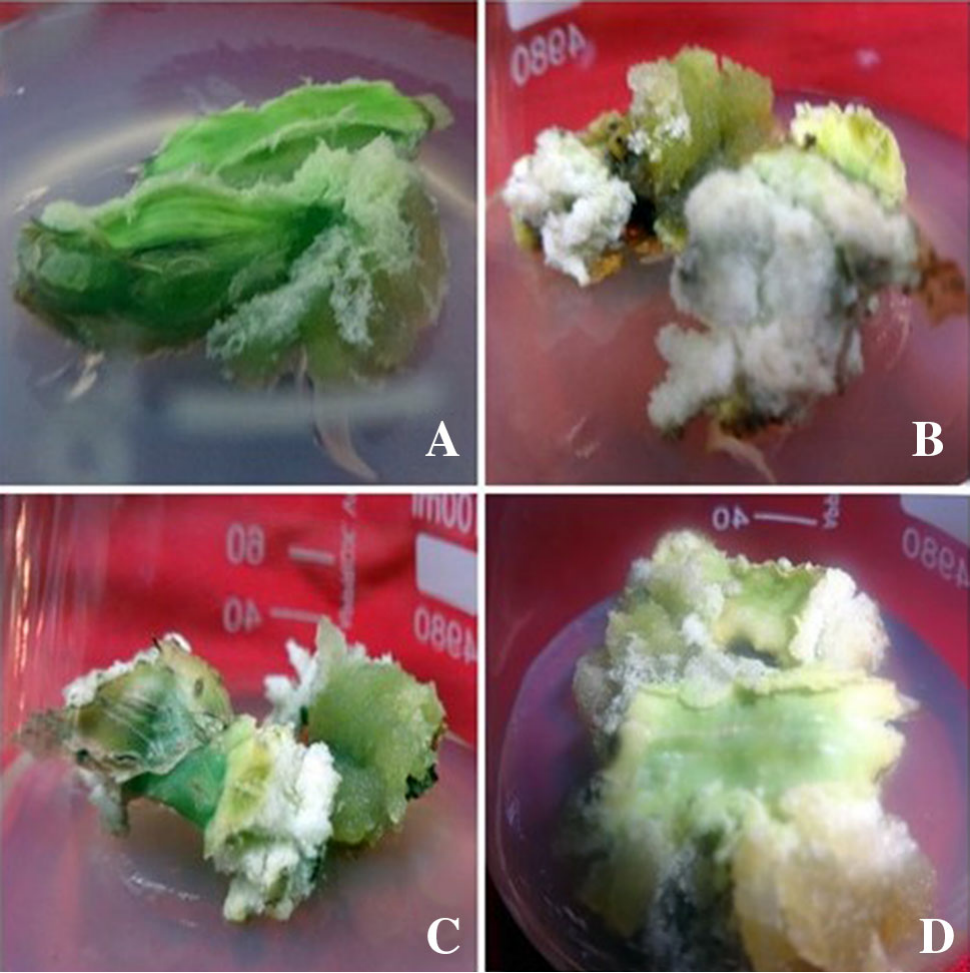
**a–d** Callus production via the culture of inter nodal segments on MS medium supplemented with IBA; **a**: Effect of IBA (0.5 mg L^−1^) on callus production after 3 weeks of inoculation; **b**: Optimum callus production on IBA (1.0 mg L^−1^) after 3 weeks of inoculation; **c**: Effect of IBA (2.0 mg L^−1^) on callus production after 3 weeks of inoculation; **d**: Stock callus production for isolation of secondary metabolites

Callus initiation was observed when inter nodal segments were inoculated on MS basal medium supplemented with various concentrations (0.5–8.0 mg L^−1^) of IBA, IAA, NAA, 2,4-D separately. Inter nodal segments when cultured horizontally on to MS medium supplemented with IBA at (0.5, 1.0, 2.0 mg L^−1^) started swelling followed by callus formation after 2 weeks of inoculation (Fig. 2a–d). The callus was green and fragile initially but after 1 week leaching of phenolic compounds was also observed, the secreted phenolic compounds retarded further growth of callus. This problem problem was overcome when the explants as well as the medium was treated with various adjuvant (PVP, activated charcoal, citric acid and ascorbic acid). When 3 weeks old callus was subcultured on MS medium fortified with IBA mg L^−1^ and PVP 0.1% (w/v) gave optimum results to control leaching and produced stock callus.

Green callus sub cultured on BAP (1.0 mg L^−1^) in combination with different cytokinin Kn (0.5–2.0 mg L^−1^), 2iP (0.5–2.0 mg L^−1^) and TDZ (0.5–2.0 mg L^−1^) and BAP (1.0 mg L^−1^) in combination with different auxin IAA (0.5–2.0 mg L^−1^) and (0.5–2.0 mg L^−1^) for indirect shoot induction from callus. No positive response revealed for indirect shoot induction from callus using any combination of hormones tested. Many combinations of cytokinins–cytokinins and auxin–cytokinins tested did not exhibited shoot.

Callus was reported from inter nodal segments in a number of plant species viz., *Tectona grandis* (Widiyanto et al. 2005), *Mentha arvensis* (Johnson et al. 2011), *Dandrocalamus asper* (Shroti et al. 2012), *Satureja hortensis* (Navroski et al. 2012), which are in favour of present results.

However, in contrast to our findings, Haque et al. (2008) reported callus induction through nodal explant in *Cucurbita maxima* and *Benincasa hispida* on MS medium supplemented with BAP + 2,4-D and BAP + NAA, respectively.

### Histology

**Fig. 3:**
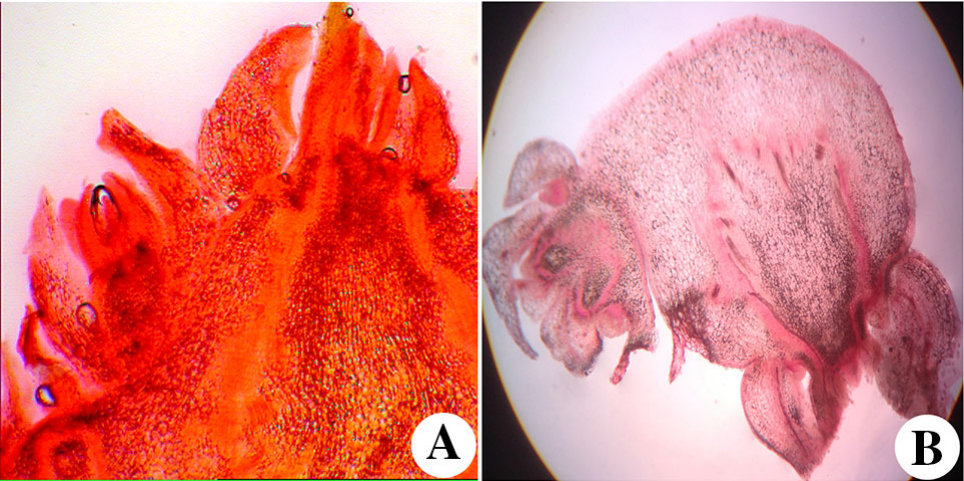
**a**, **b** Histological analysis of in vitro regenerated plantlets; **a** Histology of 2 weeks old regenerated shoots; **b** Histology of 4 weeks of regenerated shoots

Histological changes during shoot bud differentiation from nodal segments cultured on BAP and 2-iP was studied in detail. Direct differentiation of shoot buds from nodal explants was observed on BAP (1.0 mg L^−1^) supplemented in MS medium. During the first week of culture, no apparent histological changes were observed. Well-developed shoot buds with subjacent leaf primordial started appearing in the 3rd–4th week of inoculation (Fig. 3a). The origin of shoot buds was direct as indicated by the presence of vascular connections with the mother tissue. Completely developed shoot buds were connected to the nodal segments. In the cultured nodes, at a later stage of development, vertical and lateral expansion of the meristematic zone occurred (Fig. 3b). A ring of multiple shoot primordia could be observed arising directly from the base of cultured nodal segments (Fig. 3a, b).

### Molecular analysis

It is known that micropropagated tissues are easily exposed to somaclonal variations, especially during long-term cultures (Larkin and Scowcroft 1981). ISSR is the favored method used to determine the genetic stability of regenerated plants of *T. cordifolia*. The obtained band patterns were compared between randomly-selected in vitro regenerated and mother plants.

**Fig. 4:**
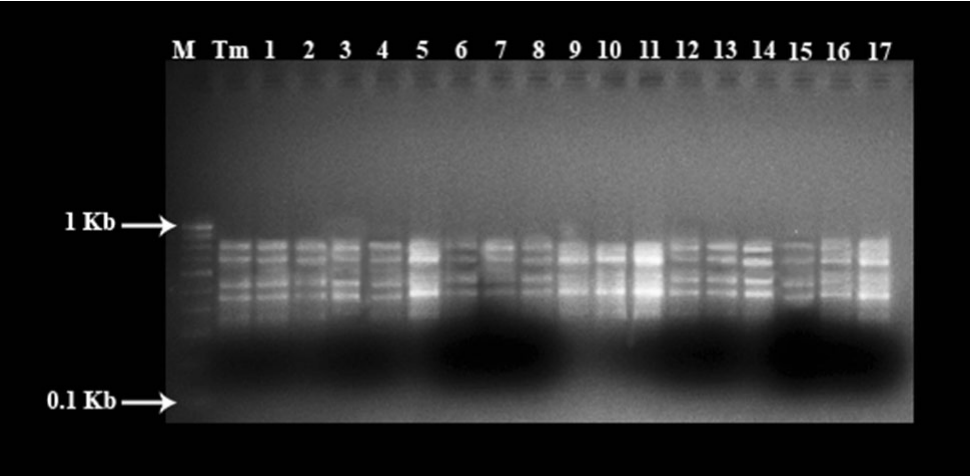
ISSR profiles of mother plant and regenerated plants of *T. cordifolia* with UBC 808 primer, Lane M ladder, lane Tm mother plant, lanes 2–18 randomly selected regenerated plants

Among 20 ISSR primers selected for the PCR amplification, 9 produced distinct and scorable bands. A total of 774 amplicons were obtained and primer UBC 808 produced highly reproducible banding pattern with 6 bands (Table 3; Fig. 4). The number of bands amplified in each ISSR primer ranged from 4 to 6.

**Table 3:** List of ISSR primers used for assessment of the regenerated and the mother plants *T. cordifolia*; their sequence, annealing temperature and number of the amplified bands

Primer	Sequence	Annealing temperature (°C)	Total bands
UBC 801	ATATATATATATATATT	33	–
UBC 802	ATATATATATATATATG	31	–
UBC 803	ATATATATATATATATC	31	–
UBC 804	TATATATATATATATAA	33	–
UBC 805	TATATATATATATATAC	31	–
UBC 806	TATATATATATATATAG	31	–
UBC 807	AGAGAGAGAGAGAGAGT	45	4
UBC 808	AGAGAGAGAGAGAGAGC	47	6
UBC 809	AGAGAGAGAGAGAGAGG	47	5
UBC 810	GAGAGAGAGAGAGAGAT	45	5
UBC 811	GAGAGAGAGAGAGAGAC	47	4
UBC 812	GAGAGAGAGAGAGAGAA	45	6
UBC 813	CTCTCTCTCTCTCTCTT	45	4
UBC 814	CTCTCTCTCTCTCTCTA	45	5
UBC 815	CTCTCTCTCTCTCTCTG	47	4
UBC 816	CACACACACACACACAT	45	–
UBC 817	CACACACACACACACAA	45	–
UBC 818	CACACACACACACACAG	47	–
UBC 819	GTGTGTGTGTGTGTGTA	45	–
UBC 820	GTGTGTGTGTGTGTGTC	47	–

The ISSR analyses revealed that the in vitro derived plants of *T. cordifolia* exhibited same banding patterns as that of mother plants, confirming that no genetic variation occurred in the DNA of in vitro regenerated plantlets. Similarly, no genetic differences were reported in regenerated plantlets of *Tylophora indica* (Sharma et al. 2014).

### Biochemical analysis

**Fig. 5:**
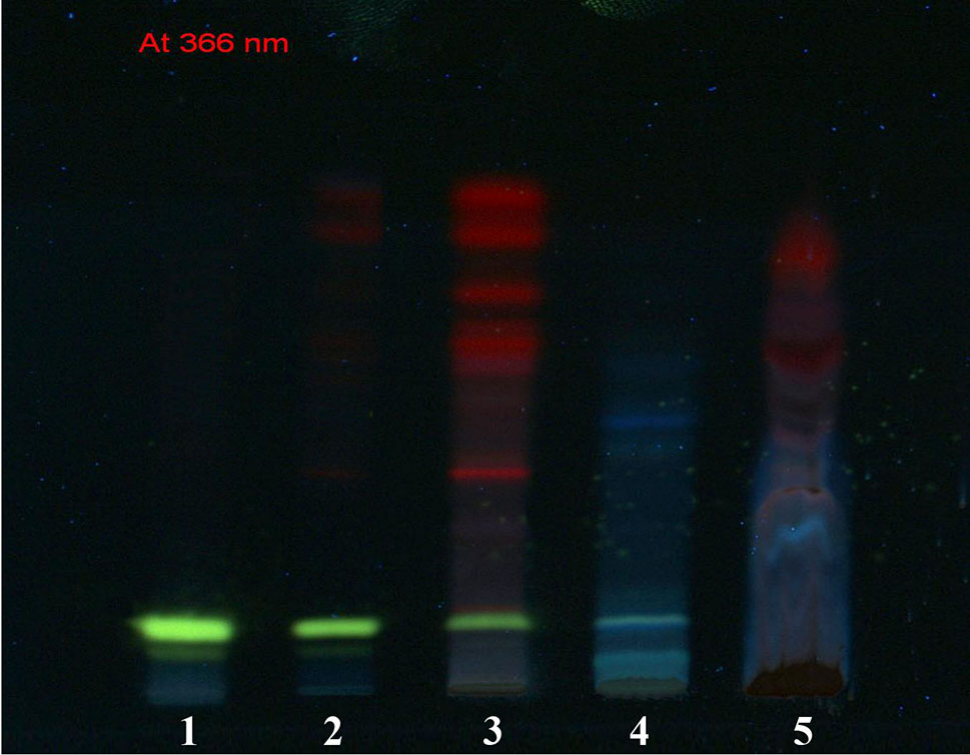
TLC chromatograph *1* Standard berberine; *2* Methanolic extract of 6 weeks old stock callus showing peak of berberine; *3* Methanolic extract of in vivo stem showing peak of berberine; *4* Methanolic extract of in vivo leaf showing peak of berberine; *5* Methanolic extract of in vivo aerial roots showing absence of berberine

TLC of different extracts (leaf, stem, aerial roots and callus) revealed that berberine was biosynthesized in different plant parts of *T. cordifolia* (Fig. 5). The concentration of berberine was higher in the methanolic extract of in vitro raised callus of *T. cordifolia* as the intensity of the bands in TLC was higher than that of the methanolic extract of leaves and stem of field-grown plants. The berberine bands were absent in the methanolic extract of aerial roots.

**Fig. 6:**
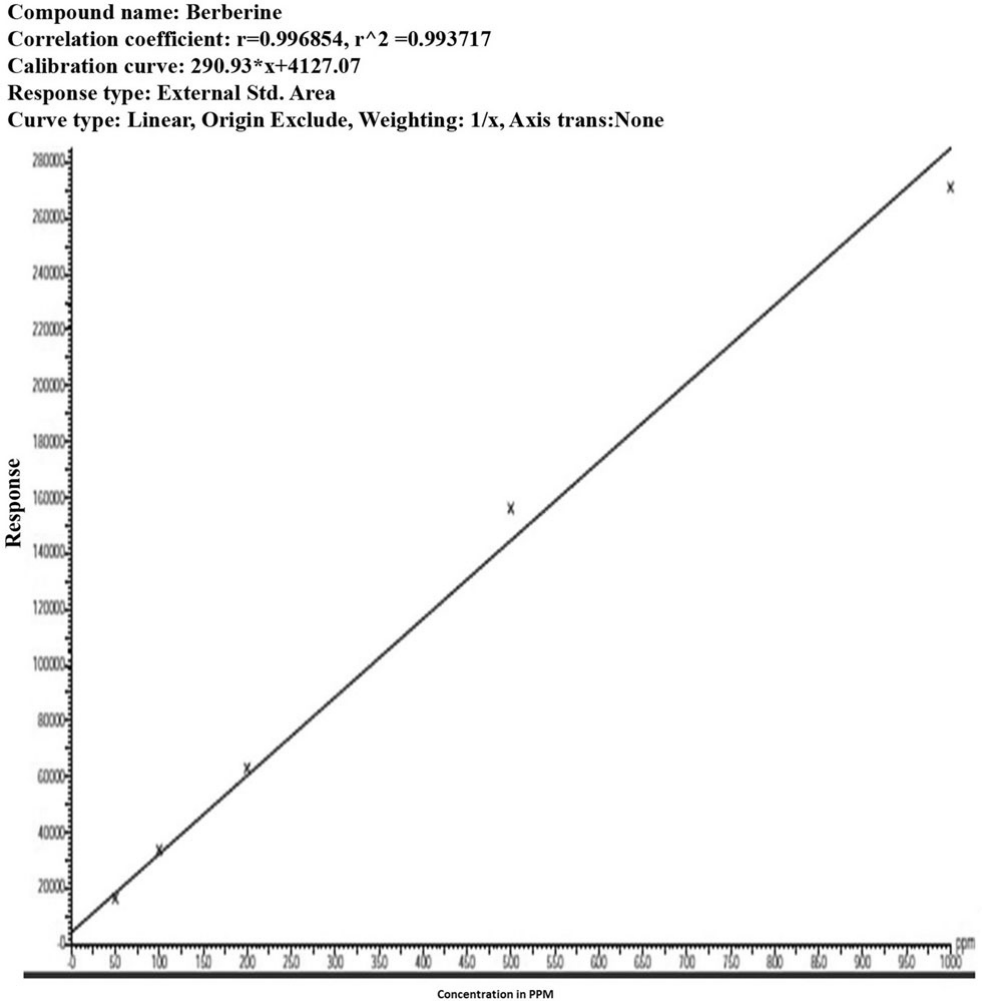
Linearity curve of standard berberine in UPLC

Further confirmation and quantification of the berberine in the various plant parts was performed by UPLC-ESI/MS positive mode. Standard sample of berberine was used to construct a calibration curve by plotting peak areas versus the amount of berberine over a range of 50–1000 ng μL^−1^ (Fig. 6). The co-elution of other components of a complex biological sample matrix with the targeted compound can be checked by means of DAD spectral data acquisition.

**Fig. 7:**
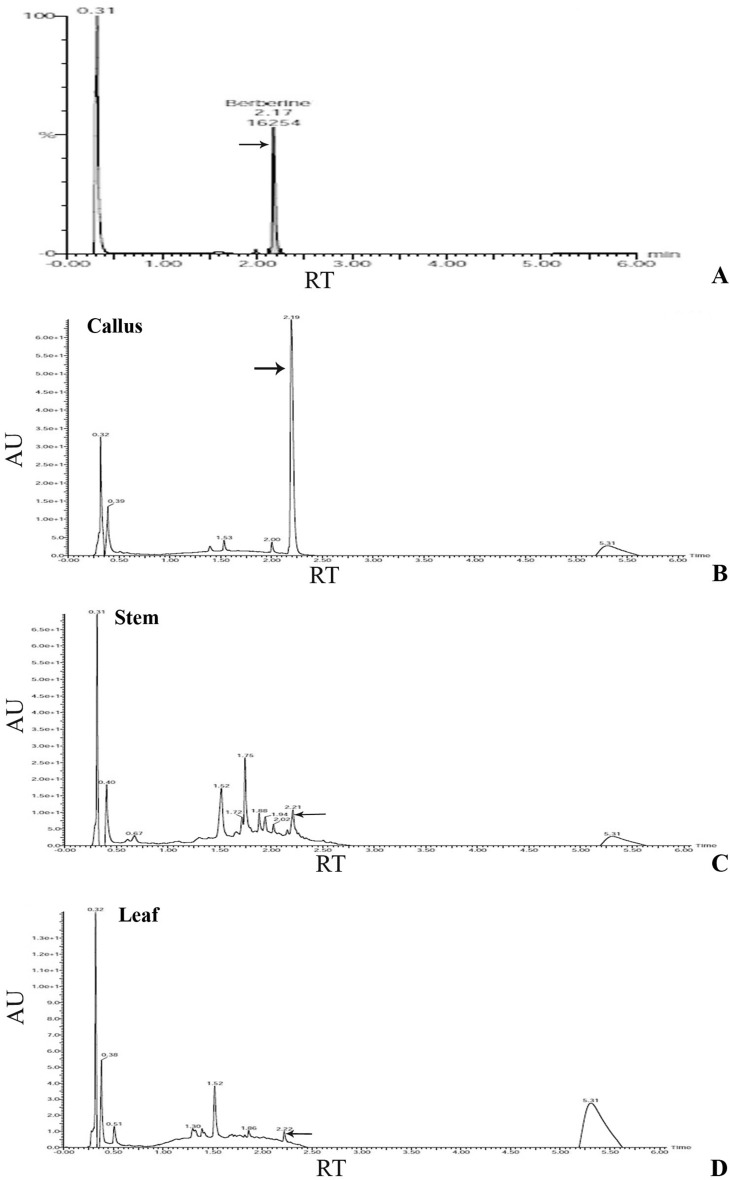
UPLC chromatogram of* 1* Standard berberine,* 2* Callus,* 3* Stem,* 4* Leaf

The identification of berberine was confirmed on the basis of retention time and absorption spectra on UV-DAD (2.20 ± 0.1 min, 266 nm) (Fig. 7). The response was linear over the tested concentration range (Table 4) with the *R* ^2^ value of 0.9937 (Fig. 6). On the basis of the retention time and absorbance spectrum, berberine was detected in the plant extracts. Quantitative UPLC analysis of the callus extract showed that maximum berberine 19.8 µg/gm dry weight was produced in in vitro callus developed by inter nodal segments (Table 4; Fig. 7b). The concentration of berberine in stem (in vivo growing) was 9.3 µg/gm while in leaf (in vivo growing) it was 8.4 µg/gm (Table 4; Fig. 7c). Similar results of plant metabolites also reported earlier by Chaodhary et al. (2015) and Jain et al. (2016).

**Table 4:** UPLC profile of *T. cordifolia* stem, leaf, aerial roots and callus

Sn	Name of sample	Type of sample	RT	Area	Height	Std. concentration	PPM	% Rec
1	MeOH	Solvent	2.16	0	18		0.0	
2	Berberine_1	Standard	2.19	16,254	487,009	50.000	43.2	86.3
3	Berberine_2	Standard	2.20	33,761	817,371	100.000	105.5	105.5
4	Berberine_3	Standard	2.16	63,083	1,251,462	200.000	209.9	104.9
5	Berberine_4	Standard	2.18	156,114	2,169,769	500.000	541.0	108.2
6	Berberine_5	Standard	2.16	271,144	2,784,344	1000.000	950.5	95.0
7	Sample_1 (leaf)	Analyte	2.19	90	2500	–	8.4	–
8	Sample_2 (stem)	Analyte	2.14	102	4536	–	9.3	–
9	Sample_3 (callus)	Analyte	2.21	1260	48,508	–	19.8	–
10	Sample_4 (aerial root)	Analyte	2.21	–	–	–	–	–

Compound name: Berberine

Correlation coefficient: *r* = 0.996854, *r*^2 = 0.993717

Calibration curve: 280.93 * x + 4127.07

Response type: external std, area

Curve type: linear, origin: exclude, weighting: 1/x, axis trans: none

**Fig. 8:**
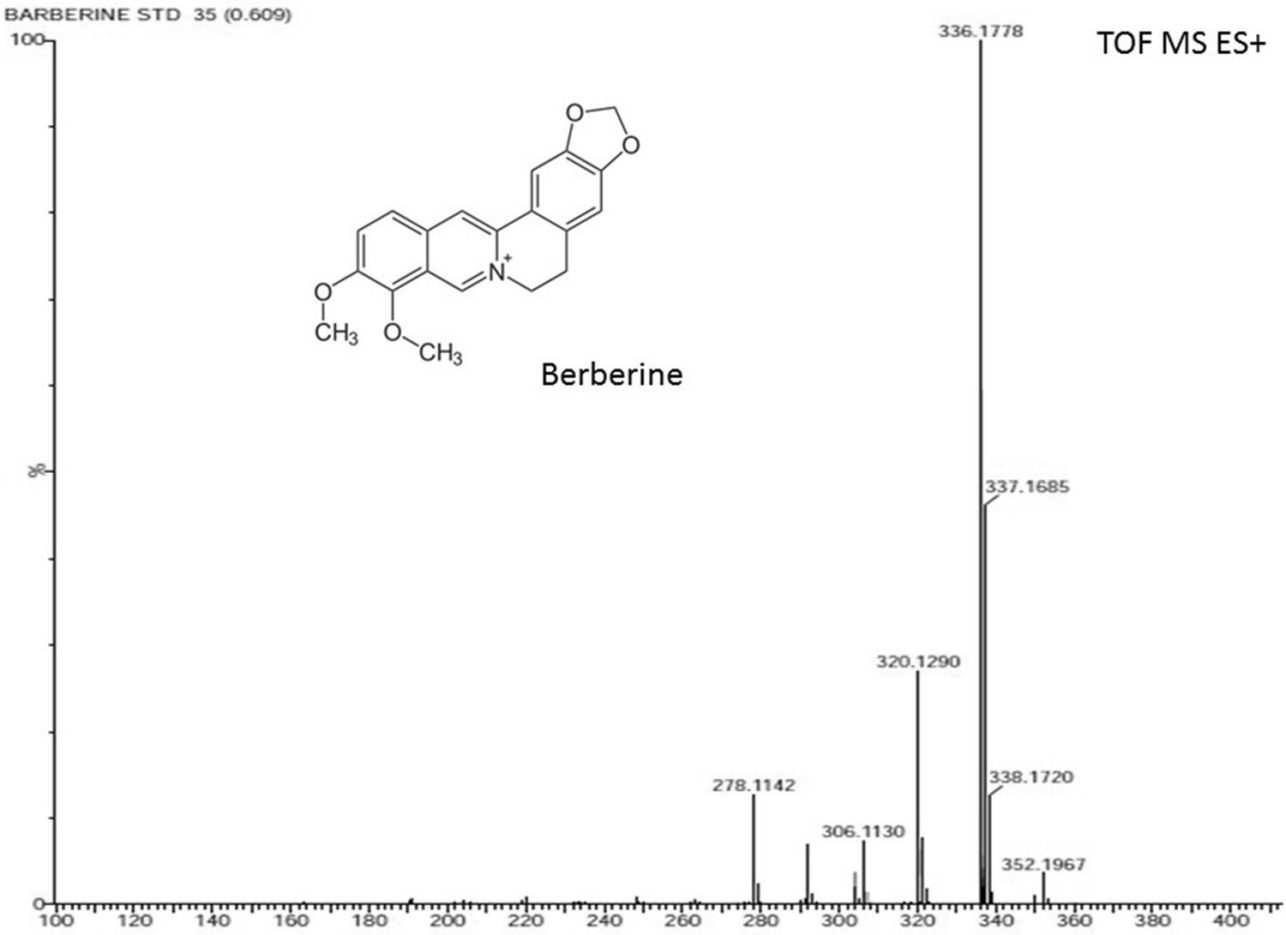
ESI–MS spectra of berberine showing molar mass of berberine at 336.178

**Fig. 9:**
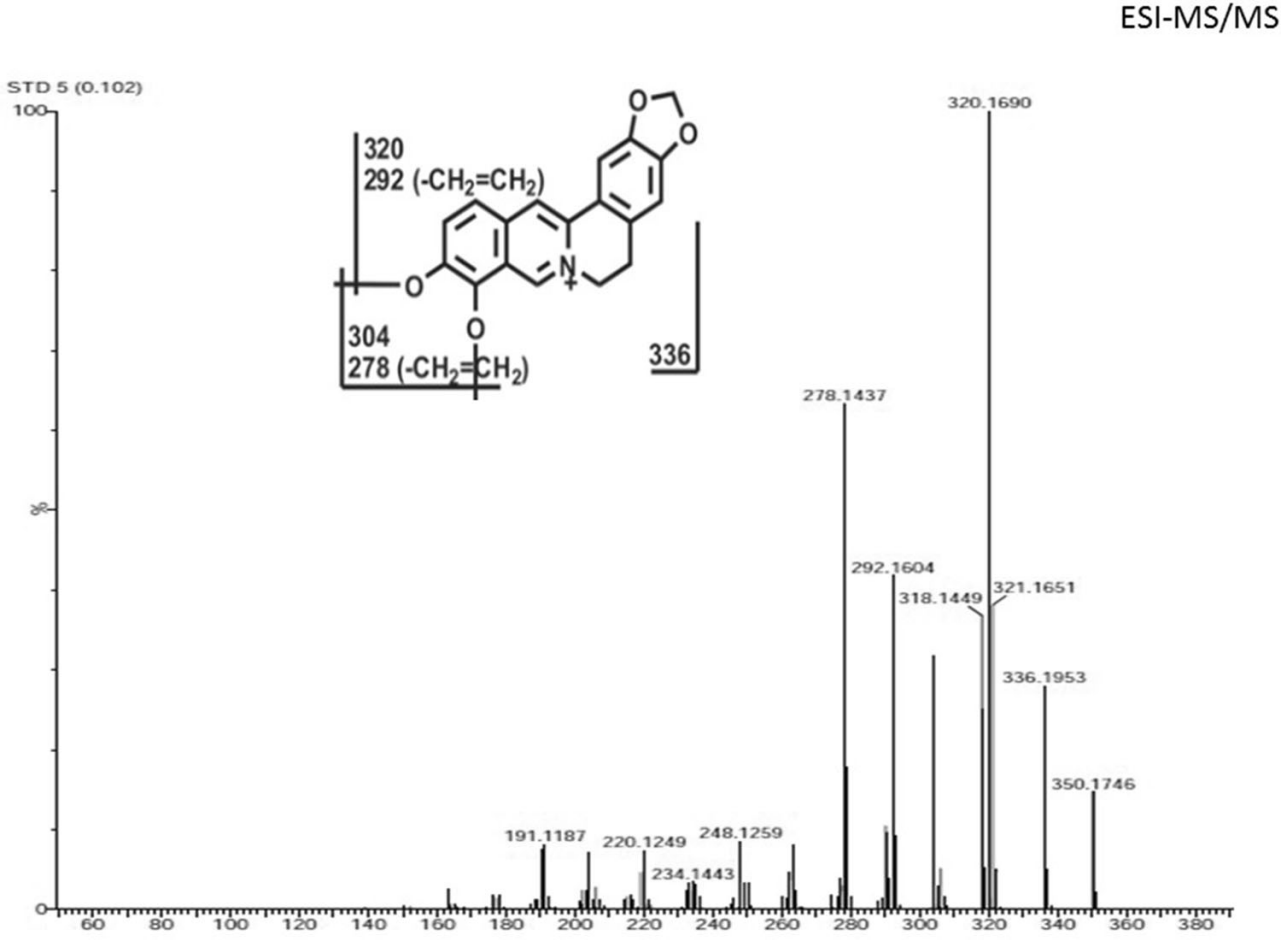
ESI–MS/MS spectra of berberine showing fragmentation pattern of berberine

For further confirmation, standard berberine and plant samples were injected into ESI–MS in continuation with UPLC for their mass spectra. LC–ESI/MS is an ideal analytical method for alkaloid analysis (Gu et al. 2002; Wu and Prior 2005) as it can distinguish between monomers and oligomers (Gu et al. 2003; Kilambi et al. 2016; Nakata et al. 2016). The berberine content of *T. cordifolia* was determined by generating reconstructed ion chromatograms (RIC) (Fig. 8). RICs were obtained by filtering data for *m/z* ratios corresponding to singly charged [M + H]^+^. The MS study of the ions allowed the detection of berberine, in *T. cordifolia*, with protonated molecular ions ([M + H]^+^) at *m/z* 336 (Fig. 8). These compounds showed similar fragmentation patterns as compared to standard berberine compound. Based on the MS data, compound was considered to be berberine composed of C_20_H_18_NO_4_^+^ elements. Both the spectra (standard and samples) showed common peaks of fragments of berberine in the form of protonated molecular ions ([M + H]^+^) at *m/z* 320, *m/z* 304, *m/z* 292, *m/z* 278 in MS/MS mode (Fig. 9). Peak at *m/z* 320, 304, 292 and 278 are the characteristic fragments of berberine which confirms presence of berberine. Berberine has already been detected in *T. cordifolia* (Srinivasan et al. 2008) and other plant species viz., *Berberis asiatica*, *Berberis aristata, Berberis lycium* (Andola et al. 2010), *Jeffersonia dubia* (Jeong and Sivanesan 2016) by using different techniques like HPLC, HPTLC etc. This is the first report of analysis of berberine from *T. cordifolia* through LCMS to the best of our knowledge.

## Materials and methods

### Plant material

Stem cuttings were collected from Nursery, University of Rajasthan, Jaipur and established in Manipal University Jaipur campus garden for regular procurement of explants for tissue culture.

Different explants viz., nodal segments having axillary bud and inter nodal segments were collected from one year old plant. All explants were surface sterilized through standard surface sterilization method.

### Culture media and growth conditions

Basal Murashige and Skoog (MS) medium was used for the study. Stock solutions of macro nutrients at 20× and 100× for micronutrients (Mo, Co, Co and Vitamins) were prepared. Beside MS salts, 3% sucrose as a carbon source and 0.8% agar–agar as a gelling agent were added. Subsequently, the nutrient medium was sterilized in autoclave at 121 °C and 15 psi pressure for 20 min.

The culture vials after inoculation were incubated at 25 ± 2 °C temperature under 16 h photoperiod with cool, white fluorescent lights (Philips India Ltd, Mumbai) of 25 μmol m^−2^ s^−1^ light intensity and 8 h dark cycle with 55 ± 5% relative humidity. All experiments were set with eight replicates and repeated at least thrice. Cultures were observed daily for the assessment of any morphological changes, if occurred in the cultures.

### Micropropagation

Mature nodal segments (1.0–1.5 cm long) were inoculated vertically on MS medium augmented with different concentration of cytokinins viz., BAP, Kn, 2-iP and TDZ (0.5–8.0 mg L^−1^) separately to proliferate axillary buds to micro shoots. 3 weeks old in vitro raised shoots developed on MS medium fortified with BAP (1.0 mg L^−1^) singly were subcultured on MS medium supplemented with BAP (1.0 mg L^−1^) in combination with Kn (0.5–2.0 mg L^−1^), 2iP (0.5–2.0 mg L^−1^) and TDZ (0.5–2.0 mg L^−1^) for maximizing the number of shoots and length as well.

8–9 cm long in vitro shoots with three to four leaves were separated from shoot cluster and transferred on varied strength of MS salt medium (¼, ½ and full) fortified with IBA, IAA and NAA (0.1–2.5 mg L^−1^) singly for in vitro root development. Phytagel (0.3%) instead of agar–agar was used as gelling agent to get quality photographs of in vitro rooting. The complete plantlets were carefully taken out from culture vials and thoroughly washed with autoclaved distilled water and planted in earthen pots having a mixture of autoclaved garden soil and cocopeat (2:1). These plantlets were covered with inverted glass beakers to maintain high humidity. The plantlets were nourished by providing few drops of MS salt solution with water and kept in plant growth chamber for hardening. Gradually, these plants were acclimatized and transferred in natural environment. The 15 days old hardened plantlets were transplanted to field.

### Callus culture

Inter nodal segments were inoculated on MS medium supplemented with various concentrations of different plant growth hormones including IBA, IAA, NAA and 2,4-D (0.5–8.0 mg L^−1^) separately for callus initiation. After one week of inoculation, explants showed swelling, curling from the cut ends and then the margins of explants turned into cluster of cells. 3–4 weeks old callus was subcultured on MS medium fortified with BAP (1.0 mg L^−1^) in combination with Kn (0.5–2.0 mg L^−1^), 2-iP (0.5–2.0 mg L^−1^) and TDZ (0.5–2.0 mg L^−1^) in combination for further organogenesis.

### Leaching of phenolics

At the time of in vitro culture, growth of culture stopped due to leaching. Explant and medium both turned brown. To reduce it explants were socked in liquid mixture of adjuvant (PVP, activated charcoal, citric acid and ascorbic acid) and were cultured on MS medium containing adjuvant (PVP 0.1% w/w). These explants gave better results than untreated explants.

### Histology

Histological changes were recorded for direct organogenesis in *T. cordifolia*. For which shoots at different developmental stages were used for histological studies. Shoots were preserved in FAA for 48 h for killing the microbes followed by fixation of the material in 70% alcohol. The fixed material was passed through dehydration and TBA-xylol series (Johanson 1940). Infiltration, embedding and blocks were prepared in paraffin wax. Serial Sections (10 μm) were cut with the help of a rotary microtome (Yorco, India) and these were fixed on the slides with the help of 4% formalin and Haupt's adhesive. The slides were dipped in pure xylene for dissolution of wax and were passed through an alcohol series. These slides were stained with 1% (w/v) safranine (Himedia, India) and again passed through an alcohol series for destaining. Finally, the sections were mounted in DPX (Merck, India) and observed under a photographic microscope (Olympus, India).

### Analysis of clonal fidelity

To assess the genetic fidelity of the regenerated plantlets of *T. cordifolia*, DNA was extracted from the leaves of 17 randomly selected in vitro regenerated plants and from the leaves of 2-year-old mother plant (MUJ campus) by CTAB method (Doyle and Doyle 1990). The quantity and quality of extracted DNA samples were estimated by comparing with uncut lambda DNA of known concentration on agarose gel (molecular biology grade, Himedia, India).

For the optimization of ISSR reactions, DNA extracted from randomly selected in vitro plants and 20 oligonucleotide primers for ISSR analysis were used for PCR amplification reaction. The optimal annealing temperature was found to vary according to base composition of the primers. 20 ISSR primers (University of British Columbia, primer set no. 9, Vancouver, Canada) were initially screened to assess clonal fidelity of *T. cordifolia.* Amplification was carried out in 20 µL reaction volume containing 2.5 µg genomic DNA as template, 2.5 µL MgCl_2_, 0.5 µL of 100 µM dNTP, 2 µL of Taq buffer B (Exclude MgCl_2_), 2.5 µL of ISSR primer, 0.35 µL of Taq Nova DNA polymerase and MilliQ water to make up the final volume. PCR amplifications were performed with initial denaturation at 94 °C for 5.0 min followed by 35 cycles of denaturation at 92 °C for 1.0 min, annealing at temperature (depending on the primer Tm) for 1.0 min, extension at 72 °C for 2.0 min with a final extension at 72 °C for 7.0 min using DNA Engine (BioRad, Germany). The PCR products were separated on 1.5% agarose gel (Himedia, India) using 100 bp and 1 kb markers (Bangalore Genei, India) as the band size standard and photographed in a gel documentation system (Bio-Rad, Germany).

### Biochemical analysis

Isolation of secondary metabolites from various plant parts (leaves, stem, aerial roots and callus) was performed using the method described by Sangwan et al. (2007). 2.0 g fresh harvested plant material (leaf, stem, aerial roots, callus) was extracted with 20 ml water and methanol in the proportion of 1:1 (v/v) in 100 ml Erlenmeyer flask on a orbital shaker at 20–30 rpm for 8 h and repeated thrice. The extracts in the solvent composition were recovered by filtration. All the three filtrates were pooled and subjected to liquid–liquid partition chromatography. The extract was treated with equal volume of *n*-hexane for 2 h in a separating funnel (250 ml) to remove the pigments and fatty acids. Meanwhile, solvents in separating funnel were mixed by vigorously stirring in every 20 min. The upper n-hexane layer was discarded and the process was repeated thrice. The defatted and depigmented extract was then pooled and evaporated to dryness at room temperature. The residue was dissolved in known volume (1 ml) of LCMS grade methanol and filtered through 0.22 μm filter (Millipore, India) prior to LCMS.

### TLC

For TLC, 10 μL of each plant extract (leaves, stem, aerial roots and callus) and standard berberine was loaded on precoated silica gel G-60 plates using automated TLC sample injector (Linomat 5, Camag) and run in a solvent system consisting of Toluene: Formic acid: Water: Ethyl Acetate (5:1:1:3). Dry the plates and visualized under TLC visualizer (Camag) at 254 and 366 nm. The development of TLC plate was done with Dragandroff reagent (Bismuth sub-nitrate 1.7 g, glacial acetic acid 20 ml, water 80 ml and 50% solution of Potassium iodide in water 100 ml. mix together and store as stock solution. 10 ml of stock, 20 ml Glacial Acetic Acid and make up to 100 ml with water gives the working solution) followed by heating at 110 °C. After derivatization TLC plate was again visualized under TLC visualizer (Camag) at 366 nm and white light. Authentic berberine in the form of berberine chloride was used as marker.

### LCMS

Chromatographic separation was performed on an Acquity UPLC system (Waters Corp., Milford, MA, USA) equipped with a PDA detector and HSS T3 column (50 mm × 1.0 mm, particle size 1.8 mm; Waters Corp.) applying the following binary gradient at a flow rate of 400 µL/min: 0–2 min, isocratic 90% A (water: formic acid, 99.9:0.1, v/v), 10% B (acetonitrile: formic acid, 99.9:0.1, v/v); 2 to 4 min, linear from 10 to 50% B; 4 to 5 min, isocratic 90% B; 5 to 6 min, isocratic 10% B. The injection volume was 10 µL (full loop injection). Eluted compounds were detected from *m/z* 100 to 1000 using a quadrupole time-of-flight (QTOF) mass spectrometer full scan (Waters Corp., Milford, MA, USA) equipped with an electron spray ion source (ESI) in positive ion modes using the following instrument settings: nebulizer gas, nitrogen, 1.6 bar; dry gas, nitrogen, 6 L/min, 190 °C; capillary, −5500 V (+4000 V); end-plate offset, −500 V; funnel 1 RF, 200 Vpp; funnel 2 RF, 200 Vpp; in-source CID energy, 0 V; hexapole RF, 100 Vpp; quadrupole ion energy, 5 eV; collision gas, argon; collision energy, 10 eV; collision RF 200/400 Vpp (timing 50/50); transfer time, 70 ms; pre-pulse storage, 5 ms; pulser frequency, 10 kHz; spectra rate, 3 Hz. Internal mass calibration of each analysis was performed by the infusion of 10 µL 10 mM Leucine Enkephalin in isopropanol: water, 1:1 (v/v), at a gradient time of 18 min using a diverter valve.

Berberine compound was characterized by UV–Vis spectra (266 nm), retention time relative to external standards, peak spiking, mass spectra, MS fragmentation patterns and spectra of isolated compounds from an in-house database and reference literature.

Data obtained from all experiments were presented as the mean ± standard error of three replications. Statistically significant differences were determined by analysis of variance (ANOVA) and the Duncan multiple range test (DMRT) at a *P* < 0.05 level of significance using SPPS.

## Conclusion

In this study, high efficiency shoot regeneration was achieved from nodal segments of *T. cordifolia*. An average 7.9 ± 0.45 shoots with highest shoot length 9.3 ± 0.48 cm and 86% response were produced. The plants were successfully grown in the field. The ISSR analysis revealed that in vitro propagation did not induce any genetic changes in the regenerated plantlets. Besides, LCMS-QToF quantification of berberine showed that relatively high concentration of berberine is evaluated in the callus, which indicates that callus can be used as potential sources of the berberine instead of wild plants.

## Abbreviations

BAP: 6-Benzylaminopurine
Kn: 6-Fufurylaminopurine
2-Ip: N6-2-Iso-pentenyl adenine
TDZ: Thidiazuron
2,4-D: Dichloroacetic acid
IAA: Indole-3-acetic acid
IBA: Indole-3-butyric acid
NAA: 1-Naphtalenacetic acid
ISSR: Inter simple sequence repeat
